# Motivating parents to protect their children from wildfire smoke: the impact of air quality index infographics

**DOI:** 10.1088/2515-7620/ad5931

**Published:** 2024-07-01

**Authors:** Catherine E Slavik, Daniel A Chapman, Hollie Smith, Michael Coughlan, Ellen Peters

**Affiliations:** 1 Center for Science Communication Research, School of Journalism and Communication, University of Oregon, United States of America; 2 Ecosystem Workforce Program, Institute for Resilient Organizations, Communities, and Environments, University of Oregon, United States of America; 3 Department of Psychology, University of Oregon, United States of America

**Keywords:** air quality, wildfire smoke, environmental health, children’s health, risk management, communications

## Abstract

*Background*. Wildfire smoke events are increasing in frequency and intensity due to climate change. Children are especially vulnerable to health effects even at moderate smoke levels. However, it is unclear how parents respond to Air Quality Indices (AQIs) frequently used by agencies to communicate air pollution health risks. *Methods*. In an experiment (3 × 2 × 2 factorial design), 2,100 parents were randomly assigned to view one of twelve adapted AQI infographics that varied by visual (table, line, gauge), index type (AQI [0-500], AQHI [1-11+]), and risk level (moderate, high). Participants were told to imagine encountering the infographic in a short-term exposure scenario. They reported worry about wildfire smoke, intentions to take risk-mitigating actions (e.g., air purifier use), and support for various exposure reduction policies. Subsequently, participants were told to imagine encountering the same infographic daily during a school week in a long-term exposure scenario and again reported worry, action intentions, and policy support. *Results*. Parents’ responses significantly differentiated between risk levels that both pose a threat to children’s health; worry and action intentions were much higher in the high-risk group than the moderate-risk group in both short-exposure (F = 748.68 p<.001; F = 411.59, p<.001) and long-exposure scenarios (F = 470.51, p<.001; F = 212.01, p<.001). However, in the short-exposure scenario, when shown the AQHI [1-11+] with either the line or gauge visuals, parents’ action intentions were more similar between moderate- and high-risk level groups (3-way interaction, F = 6.03, p = .002). *Conclusions*. These results suggest some index formats such as the AQHI—rather than the AQI—may better attune parents to moderate levels of wildfire smoke being dangerous to children’s health. Our research offers insights for agencies and officials seeking to improve current public education efforts during wildfire smoke events and speaks to the critical need to educate parents and help them act short-term and long-term to protect children’s health.

## 1. Introduction

Billions of people around the world are likely exposed to hazardous levels of air pollution from wildfires each year (Xu *et al* 2023). Reducing population exposures to wildfire smoke-related pollution is a significant public health challenge as instances of wildfire activity increase due to longer, warmer seasons created by climate change (Parisien *et al* 2023). Worse still, prolonged smoke events (lasting weeks to months) dispersed over large geographic areas are becoming more common due to the increasing intensity of wildfires (Burke *et al* 2021). Chronic exposure to wildfire smoke is not only hazardous to physical health for adults and children, but also impacts individuals’ mental health due to disruptions to normal activities (e.g., school closures, less outdoor recreation) that lead some families to shelter in their homes and force others out of their communities for extended periods of time to regions with cleaner air (Burke *et al* 2022).

Children are especially vulnerable to health effects from wildfire smoke, which include increased hospitalizations for asthma, upper respiratory infection, bronchitis, and pneumonia (Holm *et al* 2021). Their increased risk is because children often spend more time outdoors than adults and physical exertion requires them to breathe more air relative to their body weight, leading to higher smoke doses (Hauptman *et al* 2020). But not all parents are aware of wildfire smoke risks to children’s health nor how to reduce exposure (Santana *et al* 2021). Consequently, agencies and experts committed to the protection of environmental and human health have made considerable efforts to enhance and expand smoke-risk communication efforts, encouraging parents to check their local Air Quality Index (AQI) and adopt actions to protect children (e.g., limiting time outdoors, using air purifiers indoors, donning respirators) (US EPA, O 2024, American Academy of Pediatrics 2023, US CDC 2023). AQIs, of course, consider multiple air pollutants from various sources, but wildfire smoke—the current paper’s focus—contributes substantially to declines in local and regional air quality when it is present in the air and thus, smoke drives AQI values during wildfire seasons. In fact, wildfire smoke can lead to levels of fine particulate matter and ozone in some regions that equal or exceed concentrations more typically associated with urban or industrial emissions (Bourgeois *et al* 2021, Burke *et al* 2023). As a result, jurisdictions in North America with extended wildfire smoke periods rely on AQIs to indicate levels of smoke in the air (by way of fine particulate matter concentrations) and to communicate public health risks during wildfire seasons (Government of British Columbia 2023, Oregon DEQ Department of Environmental Quality 2023).

Fortunately, engagement with air quality information during wildfire smoke events, and individuals’ perceptions of risk related to wildfire smoke, appear to motivate the adoption of smoke-safe behaviors (Hano *et al* 2020, Slavik *et al* 2023). AQIs are often presented as visuals or infographics using a numeric, color-coded scale combined with text interpretations that indicate how polluted the air is at a given location. For example, in the US, values range from 0 ‘Good’ to 500+ ‘Hazardous’, and in Canada, values range from 1 ‘Low risk’ to >10 ‘Very high risk’ (Perlmutt and Cromar 2019). However, public understanding of these visuals may limit their ability to motivate people to take actions that minimize exposure (Smallbone 2012, D’Antoni 2019, Ramírez *et al* 2019). This lack of understanding poses a challenge to governments who disseminate air quality information and largely rely on citizens to check and use daily AQIs to inform decisions around what protective actions to take (e.g., staying indoors to limit exposure to wildfire smoke). In fact, in some studies, citizens have reported a desire for simpler AQI scales that are easier to understand and recall (Fish *et al* 2017, Ramírez *et al* 2019), with some finding the numbers used in AQIs—that range from 0 to 500+—to be difficult to contextualize and interpret (Marfori *et al* 2020). These user preferences are consistent with literature on numerical cognition that suggest people use a mental number line to compare values, placing smaller numbers on the left side of a continuous line and larger ones on the right to infer their magnitudes (in cultures where reading goes from left to right). However, larger numbers become more challenging to project on the number line as they are distorted more in human perception (Izard and Dehaene 2008, Landy *et al* 2013, Reyna and Brainerd 2023). Thus, people have an easier time distinguishing between smaller than larger values (Parkman 1971, Peters *et al* 2008, Boyce-Jacino *et al* 2022).

To present AQI values, agencies have designed a variety of infographics, employing various types of visuals such as emoji icons (Berger and Baylog 2023), horizontal line scales, and gauge charts using a dial, in addition to simple tables with text (Petersen 2002). Numerous prior works have discussed the benefits of using visuals to communicate about environmental and health risks. For example, effective visual aids have been found to direct people’s attention to key information, promote more precise and accurate understanding of risks (especially among the less numerate), and give rise to enduring changes in behavioral intentions that impact healthy decision making on issues such as hazardous waste and radon mitigation (Ancker *et al* 2006, Lazard and Atkinson 2015, Garcia-Retamero and Cokely 2017, Padilla *et al* 2018). However, little research has evaluated different AQI visuals to discern optimal approaches for conveying AQI information. Previous studies have focused instead on testing different types of messaging strategies such as the effects of using air quality phone alerts or providing people with information about air pollution health impacts (D’Antoni 2019, Delmas and Kohli 2020, 2021, Rosen *et al* 2021, Wu *et al* 2021). Consequently, a need exists for further research to test established strategies for effective visual communication from other domains to the comprehension of AQI information and the adoption of smoke-safe behaviors. As regions adapt to longer-lasting smoke events and changing fire regimes—including increases in prescribed burns as a mitigative wildfire measure—effective wildfire smoke risk communication is a critical factor in facilitating public adaptation (Fish *et al* 2017, Hagler *et al* 2021).

A common criticism of current air quality risk communication is that messaging can be vague. Clearer communication is needed to highlight differences in wildfire smoke exposures, health impacts, and interventions for different risk groups (Errett *et al* 2019, Wu *et al* 2021, Shellington *et al* 2022) and to address public perceptions of what makes someone ‘sensitive’ to air pollution (Johnson 2012). Messaging during ‘Moderate’ levels of air pollution may be especially prone to misinterpretation (Smallbone 2012). For instance, when the AQI reaches the ‘Moderate’ hazard category in the US, a warning message is not conveyed, and standard communications tend to describe the air quality as safe except for people who are unusually sensitive to air pollution (e.g., pregnant individuals, the elderly, children). Importantly, young children can experience health impacts from wildfire smoke at these lower risk thresholds (Hutchinson *et al* 2018), and thus, parents may especially benefit from detailed AQI guidance even at ‘Moderate’ air quality levels to increase action adoption and policy support for measures reducing children’s smoke exposures. No study to date has targeted parents specifically to test how they perceive and respond to AQI infographics during moderate- versus high-risk air pollution events.

Recognizing existing research gaps around smoke-related air quality communications, we investigated the effects of different representations of AQI information—adapted from visuals currently used by government agencies in North America—on participants’ worry, action intentions, and policy support. We recruited parents from three US states (Washington, Oregon, California) and one Canadian province (British Columbia) that frequently experience wildfires to participate in an online survey and experiment. The three visual formats selected as experimental conditions for this study were based on existing visuals from AirNow (the partnership of US Agencies responsible for disseminating air quality information) (AirNow 2023), which uses a gauge chart in the shape of a dial, and from Environment and Climate Change Canada (the Canadian agency responsible for disseminating air quality information) (Environment and Climate Change Canada 2021), which uses a line chart. In both Canada and the US, tabular infographics are also sometimes used by agencies to communicate air quality information. Although tables have been found to be useful for conveying precise values and estimates and helping users remember specific values (Hawley *et al* 2008, Schwabish 2021), research suggests that risk/benefit information may be better understood using visuals (Fagerlin *et al* 2007, Tait *et al* 2010). This may be especially true of effectively designed visuals that reduce cognitive effort and leverage heuristic processes for decoding information, for example, by using familiar visual cues (Jones 2015). As a result, we hypothesized that, relative to a table, infographics using line and gauge charts would prompt greater worry, action intentions, and policy support.

The two countries included in this study also use different types of indices, which we sought to test in our experiment; the US uses an AQI with values ranging from 0 to 500+, whereas Canada uses an AQ Health Index (AQHI) with values ranging from 0 to >10. Prior research has found that the representation of large numbers, including in the design of visual arrays containing large numbers, can hinder people’s perception and processing of numerical information, potentially affecting how people make decisions (Barth *et al* 2003, Boyce-Jacino *et al* 2022). As a result, they might rely more on text-based labels without additional thought about the true meaning of the health risk for their children. Hence, we explored potential differences between the AQI and AQHI on parents’ risk perceptions, actions intentions, and policy support.

Finally, due to the heightened health risks that sensitive populations like children face at even moderate levels of wildfire smoke (Delfino *et al* 2009, Hutchinson *et al* 2018, Garcia *et al* 2021), we also experimentally tested infographics displaying an air quality value classified as either moderate- or high-risk (in line with the AQI/AQHI hazard levels). Although we expected parents would, on average, report higher levels of worry and action intentions if they were assigned to view an infographic displaying a high-risk value versus moderate-risk value, we sought to explore whether certain types of visuals or indices would result in parents reporting high levels of these measures in the moderate-risk group as well given that exposure to moderate risk levels has been linked to respiratory diagnoses like acute bronchitis in children (Delfino *et al* 2009, Hutchinson *et al* 2018).

## 2. Materials and methods

### Study design and participant recruitment

Participants (*N* = 2,100) were recruited online between July 2023 and August 2023 to this randomized experiment with a 3x2x2 factorial design using two panel providers (Centiment and Leger Opinion). Many jurisdictions across North America experienced a significant wildfire smoke season in the summer of 2023, coinciding with the study period, due to record-breaking wildfires in Canada. To be eligible to participate, individuals had to be at least 18 years of age, have at least one child aged 18 years or younger, and check a box on a consent form.

After recruitment, both panel providers screened out any respondents who failed either of two attention check questions embedded in the survey and replaced these responses with additional participants. Both attention check questions simply asked participants to select a pre-defined option from a list (e.g., ‘This is an attention check. Please select ‘strongly disagree’ for this question.’). The providers also screened out any participants who completed the study faster than 1/3rd of the median completion time. Data from these participants was excluded by default and not stored for analysis. Data analysis was based only on those participants who met the criteria above and proceeded through the entire survey.

**Figure 1.**
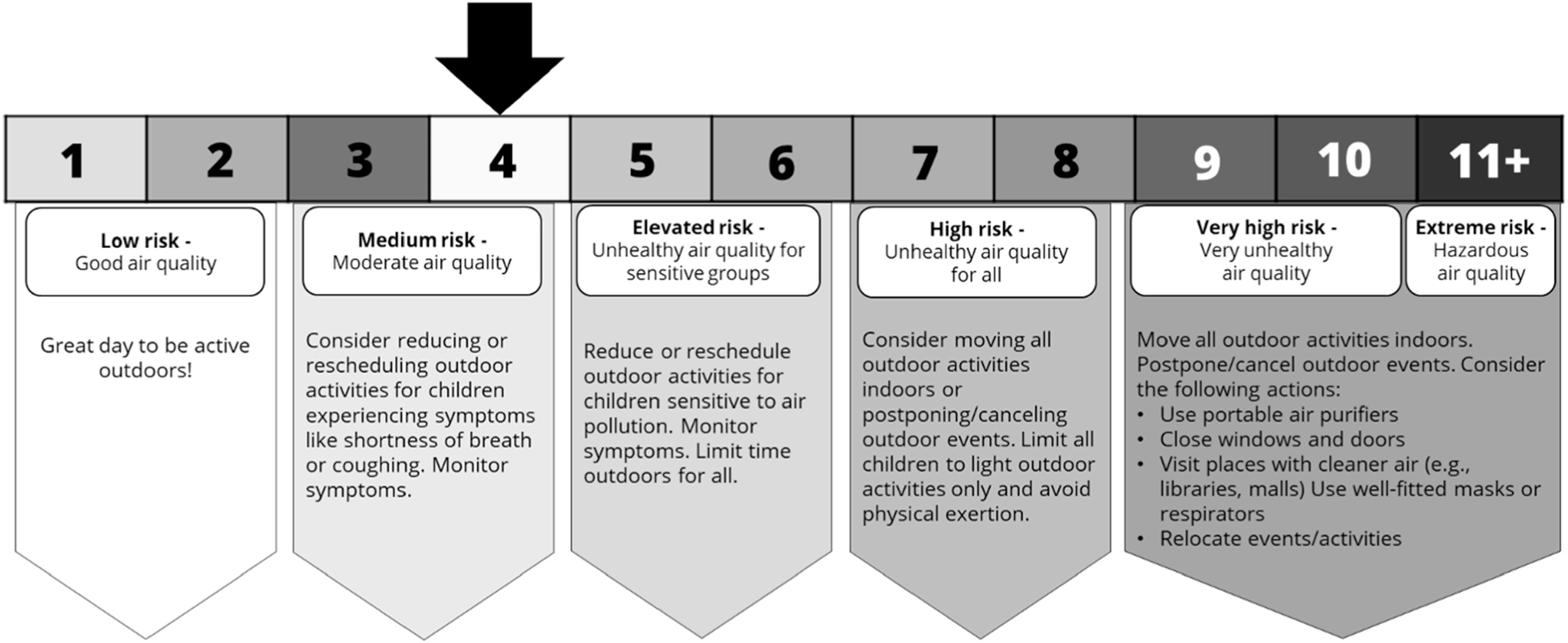
Example of one the twelve experimental infographics used displaying the line visual format with the AQHI [1-11+] index type at a moderate risk level. For the high-risk level, the arrow pointed instead at ‘8’.

Participants were randomly assigned to view one of twelve infographics that varied by visual format, numeric index type, and air quality risk level (See example in figure 1). These infographics are detailed further in section 2.3. Participants were told to imagine the air quality displayed in the infographic had occurred on a school day during a wildfire smoke event. This cue was provided so that participants were aware that the value displayed in the infographic corresponded to the levels of wildfire smoke in the air. Afterward, they were asked how worried or concerned they were about that level of air quality, how willing they would be to take certain actions to mitigate wildfire smoke exposure and whether they supported or opposed various smoke-related policy measures in a short-term exposure scenario (measures detailed further in section 2.4). Following this, participants viewed their randomly assigned infographic again with the same air quality value, and this time were told to imagine they experienced this level of air quality for an entire school week. Participants again answered the same questions about worry, action intentions, and policy support after being presented this long-term exposure scenario. No significant effects of the conditions on demographics were found using analysis of variance models, suggesting there were no failures of random assignment (See table A6).

### Study population

A balanced number of participants (i.e., parents) were recruited from Oregon (*N* = 524; 24.95%), Washington (*N* = 537; 25.57%), California (*N* = 539; 25.67%), and British Columbia (*N* = 500; 23.81%). Participants had an average age of 39.24 (*SD* = 8.92). Gender identity was assessed with response options for women (*N* = 1,996; 56.95%), men (*N* = 875; 41.67%), transgender (*N* = 2; 0.1%), non-binary/non-conforming (*N* = 21; 1%), other (*N* = 3; 0.14%), and prefer not to respond (*N* = 3; 0.14%). Educational attainment was measured using slightly different measures for the USA and Canadian samples. For parity, we rescored the education measure into a binary indicator of whether participants had a bachelor’s degree or higher (*N* = 959; 45.67%) or less than a bachelor’s degree (*N* = 1,141; 54.33%). When asked how many children under 18 they were the parent/guardian of, nearly half had 1 (*N* = 946; 45.05%), followed by 2 (*N* = 798; 38%), 3 (*N* = 251; 11.95%) and 4 (*N* = 77, 3.67%). Less than 1% had 5 children (*N* = 17; 0.81%) and six or more children (*N* = 11; 0.52%). Participants were asked to indicate which health conditions they had with response options for seven categories of health conditions: asthma, chronic obstructive pulmonary disease, hypertension or high blood pressure, other heart-related disease, type II diabetes/metabolic syndrome/obesity, allergies related to the upper respiratory tract, eyes, and ears, and/or another chronic disease. Participants could select more than one option and were asked this question about both them and their child(ren). When answering for themselves, 52.05% (*N* = 1,093) of the sample had at least one of the conditions listed. When asked about their children, 40.24% (*N* = 845) selected at least one option. When combined, 59.19% (*N* = 1,243) of the sample either had a health condition themselves or had a child with a condition.

### Experimental stimuli

**Figure 2.**
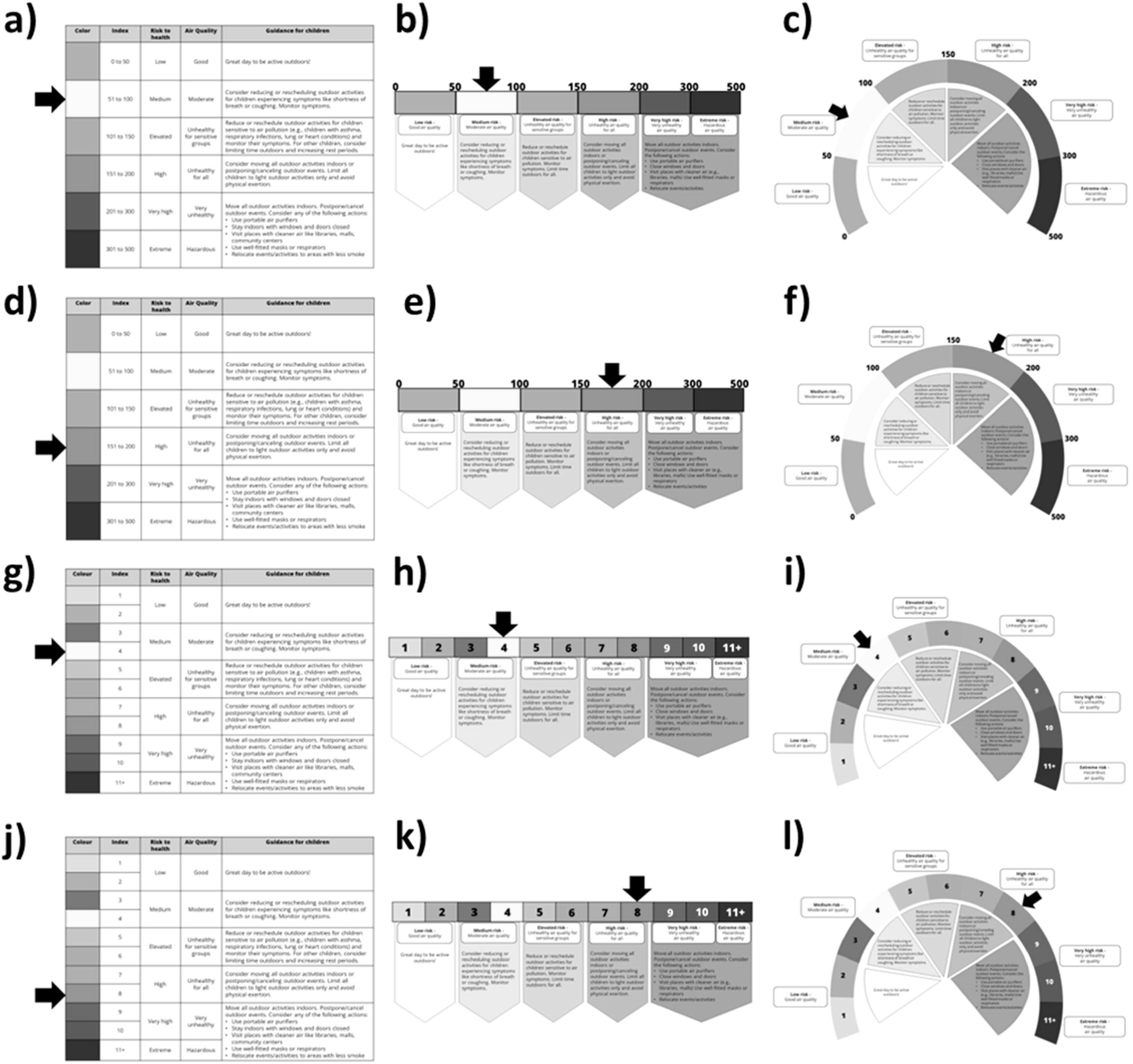
Infographics used in the experiment following a 3 × 2 × 2 factorial design, varying by visual format, index type, and risk level depicted. (a) table visual with AQI [0-500] at moderate risk. (b) Line visual with AQI [0-500] at moderate risk. (c) Gauge visual with AQI [0-500] at moderate risk. (d) table visual with AQI [0-500] at high risk. (e) Line visual with AQI [0-500] at high risk. (f) Gauge visual with AQI [0-500] at high risk. (g) table visual with AQHI [1-11+] at moderate risk. (h) Line visual with AQHI [1-11+] at moderate risk. (i) Gauge visual with AQHI [1-11+] at moderate risk. (j) table visual with AQHI [1-11+] at high risk. (k) Line visual with AQHI [1-11+] at high risk. (l) Gauge visual with AQHI [1-11+] at high risk.

This study experimentally tested twelve infographics adapted from visuals currently used by agencies in the US (AQI) and Canada (AQHI) to communicate wildfire smoke risks. These varied by visual format (table, line, gauge), numeric index type (US AQI [0-500], Canadian AQHI [1-11+]), and air quality risk level (moderate, high). An overview of all twelve experimental stimuli can be found in figure 2. Participants assigned to view a 0-500 scale at a moderate-risk level were told to imagine that the air quality level on a school day was 75 during a wildfire smoke event, while those in the high-risk group were told the air quality level was 175. Participants assigned to view a 1-11+ scale at a moderate-risk level were told to imagine that the air quality level on a school day was 4 during a wildfire smoke event, while those in the high-risk group were told the air quality level was 8. Since the risk categories of the US and Canadian scales do not exactly correspond in reality (Gladson *et al* 2022), these air quality values were selected to roughly represent exposure to similar concentrations of fine particulate matter and ensure that the distance between risk categories would appear visually similar for participants in either the AQI or AQHI groups.

### Dependent measures

A two-item composite measure assessed participants’ worry about wildfire smoke risk in general and the health impacts of wildfire smoke in particular. These questions were asked in specific reference to the experimental condition that they were in and were asked twice in the survey (once for the short-term exposure scenario and once for the long-term exposure scenario). Responses could range from 1 (*not at all worried or concerned*) to 7 (*very worried or concerned*). The two items were highly correlated in both the short-exposure context (*M* = 4.82, *SE* = 0.04), *r* = 0.87, *p* < 0.001, and the long-exposure context (*M* = 5.37, *SE* = 0.03), *r* = 0.88, *p* < 0.001.

Action intentions were measured by asking participants how willing they would be to carry out different actions if they experienced the air quality level due to wildfire smoke depicted in the short- and long-exposure scenarios. Five actions were assessed including: ‘check air quality levels in your area before going outside’, ‘cancel or reschedule outdoor activities’, ‘relocate outdoor activities indoors or to areas with better air quality’, ‘keep windows and doors closed to keep smoke out,’ and ‘use a portable HEPA air purifier to improve indoor air quality’. These items were measured on 5-point scales ranging from 1 (*not at all*) to 5 (*a great deal)*. The five items were highly correlated at both the short-exposure (*M* = 3.84, *SE* = 0.02) and long-exposure (*M* = 4.17, *SE* = 0.02) measurement points, with a Cronbach’s alpha of 0.89 in the short-term setting and alpha = 0.90 in the long-term setting.

Policy support was measured using three items which asked participants to indicate how much they would support actions taken to limit children’s exposure to wildfire smoke during unhealthy air quality days. These included: ‘moving outdoor events or activities indoors’, ‘postponing outdoor events to a later date with better air quality’, and ‘moving outdoor events to areas with better air quality’. Each was scored on a 1 (*extremely opposed*) to 6 (*extremely supportive*) scale. These items formed reliable composite indices in the short-exposure scenario (Cronbach’s alpha = 0.87) and the long-exposure scenario (Cronbach’s alpha = 0.90). Participants were, on average, in support of these measures both in the short- (*M* = 5.03, *SE* = 0.02) and long-exposure (*M* = 5.06, *SE* = 0.02) scenarios.

Support for school or childcare closure was measured by asking participants how much they would support a ‘school or childcare closure’ to limit children’s exposure to wildfire smoke during unhealthy air quality days. This question was also scored on a 1 (*extremely opposed*) to 6 (*extremely supportive*) scale. Unlike the other three policy items described above, support was, on average, lower for this measure in both short- (*M* = 4.36, *SE* = 0.03) and long-exposure (*M* = 4.35, *SE* = 0.03) scenarios. Removing the school closure measure from the policy support composite improved the Cronbach’s alpha measure of reliability for the policy support composite in both short-term and long-term scenarios. Thus, it was analyzed separately from the other three policy items.

The dependent measures were all moderately-to-strongly positively correlated with one another. The online appendix contains the full correlation matrix for these measures (table A29). A survey codebook and other study materials are available online for download (Slavik and Chapman 2024).

### Analyses

To form our dependent measures, we created composites by averaging together the variables as described in section 2.4. For measures with only two items, we checked the Pearson *r* correlation coefficient for the association between the items. For measures with more than two items, we examined the Cronbach’s alpha as a measure of scale reliability. Cronbach’s alpha values can range from 0 to 1 with values above 0.70 generally considered to reflect adequate scale reliability (Cronbach 1951). For each dependent measure, separate factorial analysis of variance (ANOVA) models were fit to the data using a 2 (index type: AQI versus AQHI) x 2 (risk level: moderate versus high) x 3 (visual format: table versus line versus gauge) design. In each model, each factor was converted into a set of summed contrast codes and their main effects and interactions were entered into the model simultaneously. These models test whether there are significant effects of each factor (e.g., risk level) independently, as well as whether interaction effects exist between the factors. Analysis of variance models of this kind are common for examining effects in factorial experimental designs (‘Analysis of Variance’ 2008) and statistical significance is determined using the conventional p < 0.05 threshold. For each model, we also calculated the *R* ^*2*^ value, which quantifies the proportion of variance in the outcome explained by the variables entered in the model (Miles 2014). *R* ^*2*^is calculated using the standard formula of 1 - (residual sum of squares/total sum of squares). We also calculated partial eta^2^ values for each factor in the model, which is an effect size measure quantifying the proportion of variance explained by each factor (for calculation and further information see: Richardson (2011)). We report the *R* ^*2*^and partial eta^2^ values in the online appendix along with the full statistical results of each model summarized in the main text.

Analyses were performed using R version 4.3.1 (R Core Team 2023). The lm function in base R was used to fit the model, and the Anova function from the *car* package (Fox and Weisberg 2019) was used to derive the ANOVA results. Type III sums of squares were used in the calculation of the ANOVA results as well as the partial eta-squared values. *R* ^*2*^ was calculated using the r2 function in the *performance* package (Lüdecke *et al* 2021). Partial eta squared was calculated using the eta_squared function from the *effectsize* package (Ben-Shachar *et al* 2020). The emmeans function from the *emmeans* package was used to derive the estimated marginal means and the pairs function was used to compute the pairwise comparisons (Lenth n.d.). Results plots were generated using ggplot from the *ggplot2* package (Wickham *et al* n.d.).

## 3. Results

### Wildfire smoke worry: Short-term and long-term exposure scenarios

#### 3.1. Short-exposure

Results of an ANOVA (See table A1) indicated significant main effects of visual format, index type, and risk level on parents’ worry (measured using a 7-point scale ranging from 1=Not at all worried to 7 = Very worried) about wildfire smoke during the short-exposure scenario. Of the three visual formats tested in this study, and counter to our hypothesis, the table (*M* = 4.93, *SE* = 0.05) elicited somewhat more worry than the line (*M* = 4.85, *SE* = 0.05) or the gauge (*M* = 4.73, *SE* = 0.05) visual formats. Pairwise comparisons showed that only the table and gauge visuals significantly differed from one another, *t* (df = 2088) = 2.67, *p* = 0.008. Neither table nor gauge visuals differed significantly from line visuals, respectively, *t* (*df* = 2088) = 0.99, *p* = 0.321 and *t* (df = 2088) = 1.68, *p* = 0.093.

**Figure 3.**
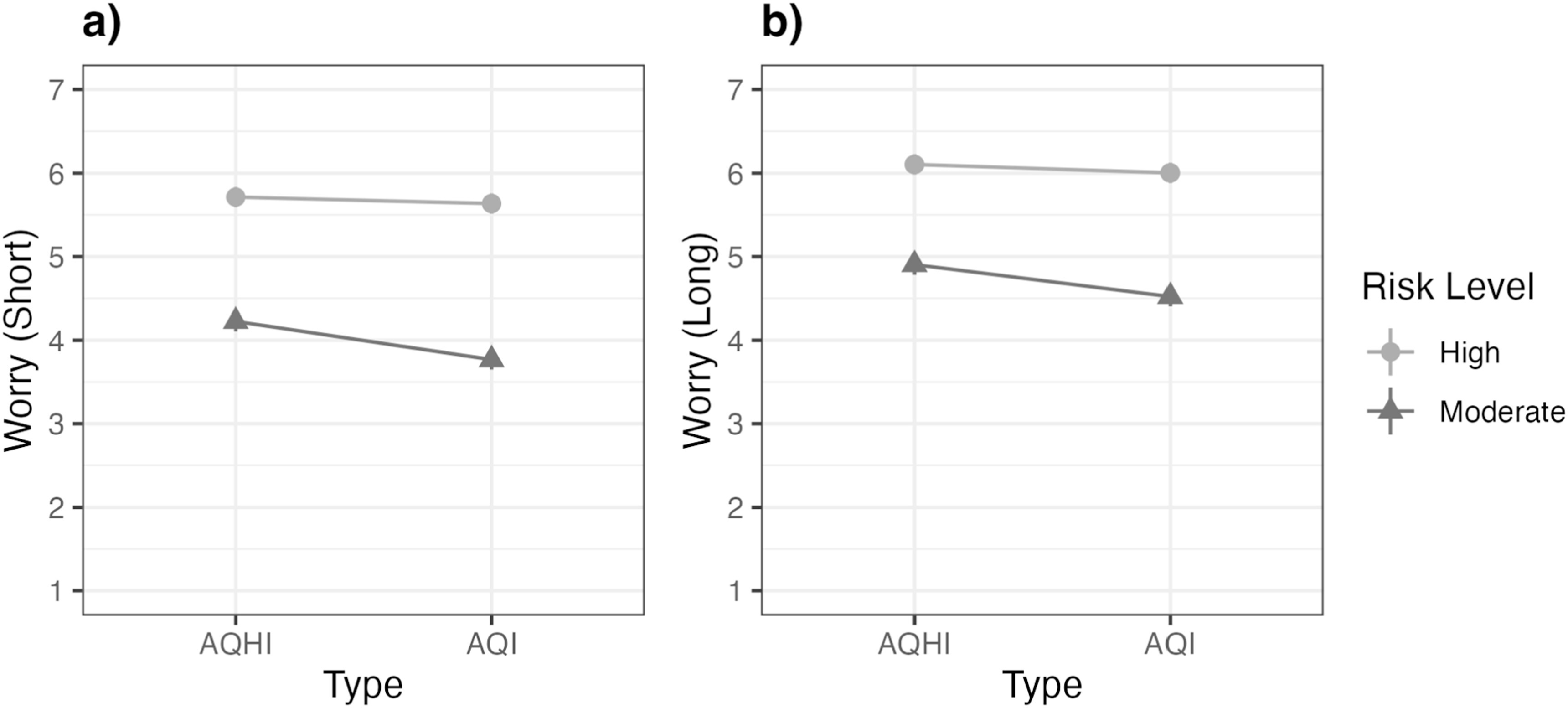
Post hoc comparison of wildfire smoke worry across the index type and risk level in the: (a) Short-term exposure scenario, and (b) Long-term exposure scenario. Worry was assessed on 7-point scales (1 = Not at all worried to 7 = Very worried).

**Figure A1.**
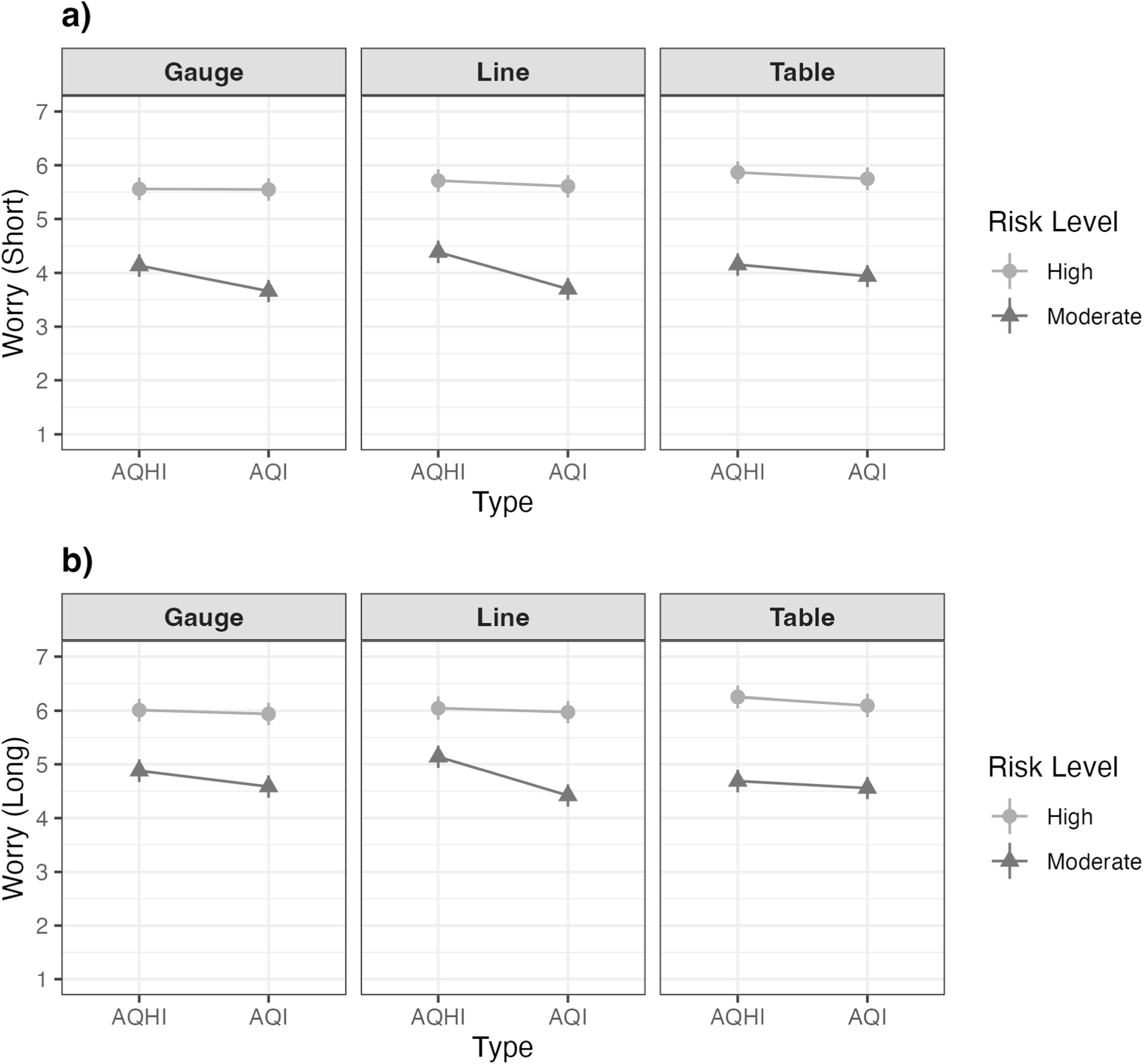
Post hoc comparison of wildfire smoke worry across the visual format, index type and risk level. (a) Short-term exposure scenario. (b) Long-term exposure scenario.

Of the two indices tested, the AQHI [1-11+] elicited greater worry (*M* = 4.97, *SE* = 0.04) than the AQI [0-500] (*M* = 4.70, *SE* = 0.04), *t* (*df* = 2088) = 4.37, *p* < 0.001. Additionally, when examining the effects of moderate- versus high-risk levels of wildfire smoke on parents’ worry, we found that infographics displaying the high-risk smoke level elicited greater worry (*M* = 5.67, *SE* = 0.04) than the moderate-risk level *(M* = 4.00, *SE* = 0.04*), t* (df = 2088) = 27.36, *p* < 0.001. However, the main effects of the risk level and index type were qualified by a significant 2-way interaction (figure 3(a)). In both the moderate- and high-risk level infographic groups, the AQHI [1-11+] elicited greater worry than the AQI [0-500], though the difference between the indices was significant in the moderate-risk group, *t* (*df* = 2088) = 5.33, *p* < 0.001, and not in the high-risk group, *t* (*df* = 2088) = 0.89, *p* = 0.373. Thus, in moderate-risk contexts, the AQHI [1-11+] infographics elicited greater worry than infographics displaying the AQI [0-500]. In high-risk contexts, the indices performed similarly. No interaction effects emerged with visual format (e.g., table versus line) (See figure A1 in the Appendix).

#### 3.2. Long-exposure

In the long-exposure scenario, analyses revealed main effects of risk level and index type, but not visual format, on wildfire smoke worry (measured using the same 7-point scale) (See table A1). As in the short-exposure scenario, the high-risk infographics elicited greater worry (*M* = 6.05, *SE* = 0.04) than the moderate-risk infographics (*M* = 4.71, *SE* = 0.04), t (*df* = 2088) = 21.69, *p* < 0.001. The AQHI [1-11+] again also elicited greater worry (*M* = 5.50, *SE* = 0.04) than the AQI [0-500] (*M* = 5.26, *SE* = 0.04), *t* (*df* = 2088) = 3.91, *p* < 0.001. These main effects were similarly qualified by a 2-way interaction between risk level and index type (figure 3(b)). The AQHI [1-11+] elicited greater worry than the AQI [0-500] in the moderate-risk group, *t* (df = 2088) = 4.40, *p* < 0.001, but not in the high-risk group; in this group, index type did not elicit different levels of worry, *t* (*df* = 2088) = 1.15, *p* = 0.252. No interaction effects emerged with visual format (See figure A1 in the Appendix).

### Action intentions: short-term and long-term exposure scenarios

#### 3.3. Short-exposure

Results of the ANOVA (See table A2) indicated significant main effects of visual format, index type, and risk level on parents’ action intentions (measured using a 5-point scale ranging from 1 = Not at all to 5 = A great deal) during the short-exposure scenario. These results closely mirrored the results for parents’ worry about wildfire smoke. Across the three visual formats, participants who had viewed the table (*M* = 3.90, *SE* = 0.04) and line (*M* = 3.88, *SE* = 0.04) visuals were the most willing to take actions to reduce their wildfire smoke exposure; intentions did not differ significantly between these two visual formats *t* (*df* = 2087) = 0.30, *p* = 0.767. Action intentions were somewhat lower among those who had viewed the gauge visual (*M* = 3.76, *SE* = 0.04), and significantly differed from those who had viewed the table, *t* (*df* = 2087) = 2.66, *p* = 0.008, and the line visual, *t* (*df* = 2087) = 2.36, *p* = 0.018.

In examining trends by index type, we found those who had viewed the AQHI [1-11+] reported greater action intentions (*M* = 3.93, *SE* = 0.03) than those who had viewed the AQI [0-500] (*M* = 3.77, *SE* = 0.03), *t* (df = 2087) = 4.03, *p* < 0.001. Results by risk level showed that the high-risk infographics elicited greater action intentions (*M* = 4.26, *SE* = 0.03) than the moderate-risk infographics (*M* = 3.43, *SE* = 0.03), *t* (*df* = 2087) = 20.29, *p* < 0.001. These results were again qualified by a 2-way interaction between risk level and index type. In the moderate-risk group, those who had viewed the AQHI [1-11+] reported higher action intentions than those with the AQI [0-500], *t* (*df* = 2087) = 5.19, *p* < 0.001. However, in the high-risk group, the AQHI [1-11+] and AQI [0-500] performed equally well at eliciting action intentions, *t* (*df* = 2087) = −0.54, *p* = 0.591.

**Figure 4.**
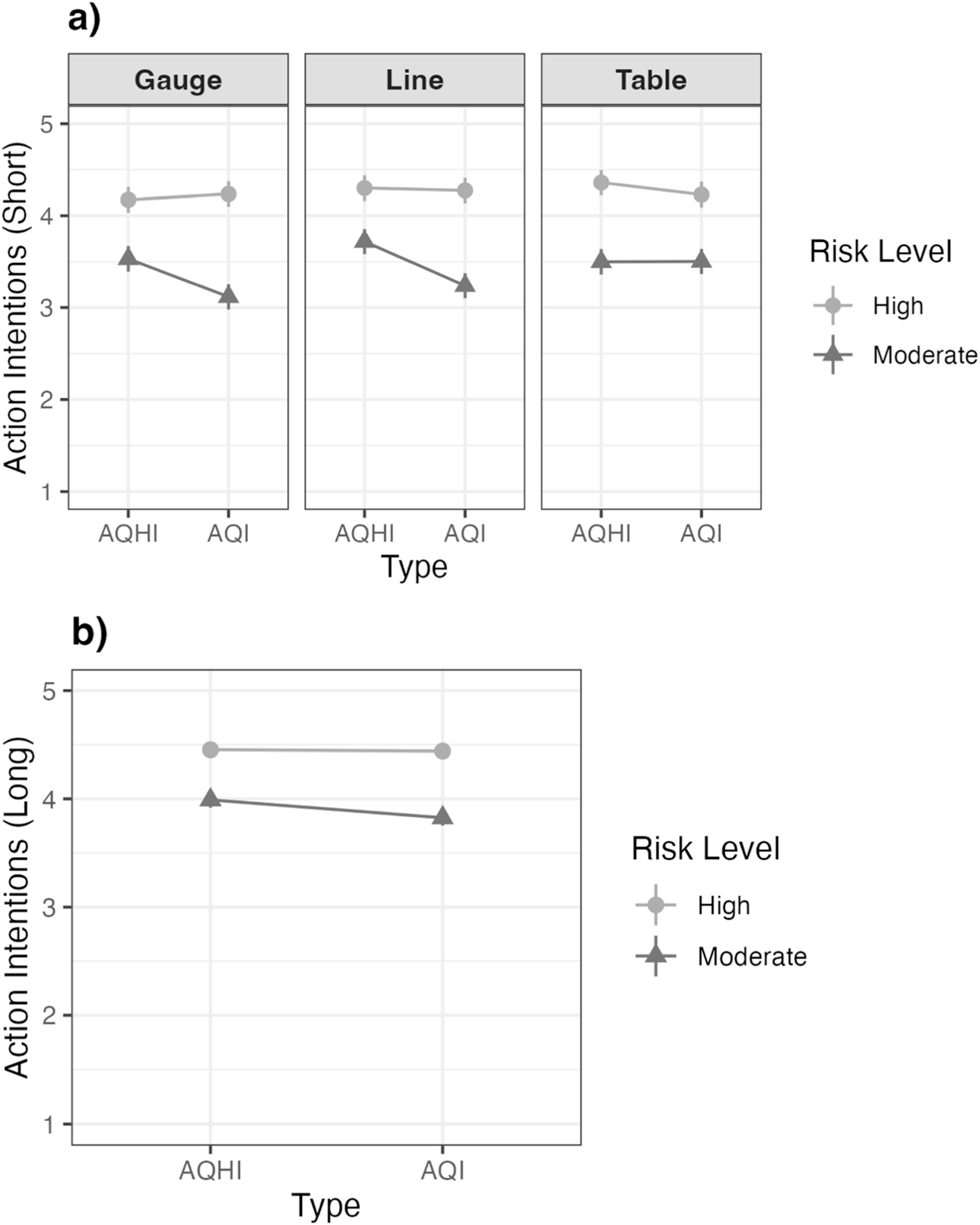
Post hoc comparison of willingness to take actions reducing wildfire smoke exposure in the: (a) Short-term exposure scenario across visual format, index type and risk level, and in the (b) Long-term exposure scenario across index type and risk level. Willingness was assessed on 5-point scales (1 = Not at all to 5 = A great deal).

In contrast to the results for worry, there was also evidence of a 3-way interaction for action intentions (figure 4(a)). Among those who had viewed the table, the AQHI [1-11+] and AQI [0-500] performed similarly in the high-risk groups (*t* (*df =* 2087) = 1.30, *p* = 0.194) and in the moderate-risk groups (*t* (*df* = 2087) = 0.04, *p* = 0.971). In other words, the 2-way interaction between index type and risk level was not present among those who had viewed the table visual. In contrast, in the line and gauge visual groups, the previously described 2-way interaction was present. Specifically, among those who had viewed the line and moderate-risk infographic, the AQHI [1-11+] elicited greater action intentions than the AQI [0-500], *t* (*df* = 2087) = 4.88, *p* < 0.001, but not in the high-risk group, *t* (*df* = 2087) = 0.26, *p* = 0.794. Among those who had viewed the gauge and moderate-risk infographic, the AQHI [1-11+] also elicited greater action intentions than the AQI [0-500], *t* (*df* = 2087) = 4.13, *p* < 0.001, but not in the high-risk group, *t* (*df* = 2087) = 0.63, *p* = 0.530. On average, parents viewing the line visual in combination with the AQHI reported the highest action intentions among those in the moderate-risk group (*M* = 3.72, *SE* = 0.07) (See table A3).

#### 3.4. Long-exposure

**Figure A2.**
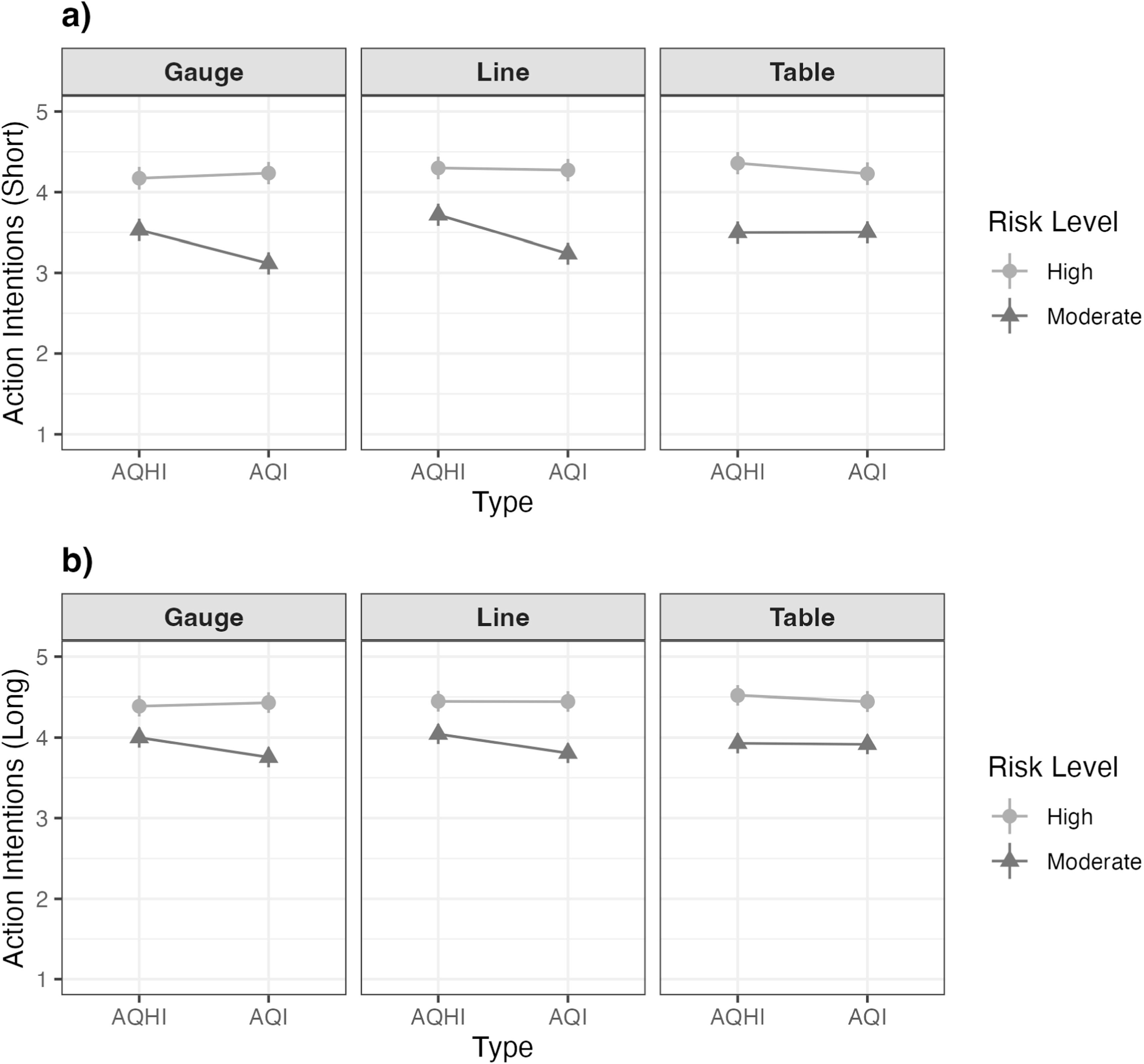
Post hoc comparison of willingness to take actions reducing wildfire smoke exposure across the visual format, index type and risk level. (a) Short-term exposure scenario. (b) Long-term exposure scenario.

In the long-exposure scenario, results for action intentions (measured using the same 5-point scale) mimicked those of worry. Analyses again revealed main effects of risk level and index type, but not visual format, on parents’ willingness to take actions to reduce wildfire smoke exposure (See table A2). As in the short-exposure scenario, the high-risk infographics elicited greater action intentions (*M* = 4.45, *SE* = 0.03) than the moderate-risk infographics (*M* = 3.91, *SE =* 0.03), t (*df* = 2088) = 14.56, *p* < 0.001. The AQHI [1-11+] again elicited greater action intentions (*M* = 4.22, *SE* = 0.03) than the AQI [0-500] (*M* = 4.13, *SE* = 0.03), t (*df* = 2088) = 2.40, *p* = 0.017. A significant 2-way interaction between risk level and index type was observed as before (figure 4(b)). Among those who had viewed the moderate-risk infographics, the AQHI [1-11+] elicited greater action intentions than the AQI [0-500], *t* (df = 2088) = 3.18, *p* = 0.002, but not in the high-risk group, *t* (df = 2088) = 0.24, *p* = 0.814. No three-way interaction emerged (See figure A2 in the Appendix).

### Policy support: Short-term and long-term exposure scenarios

**Figure A3.**
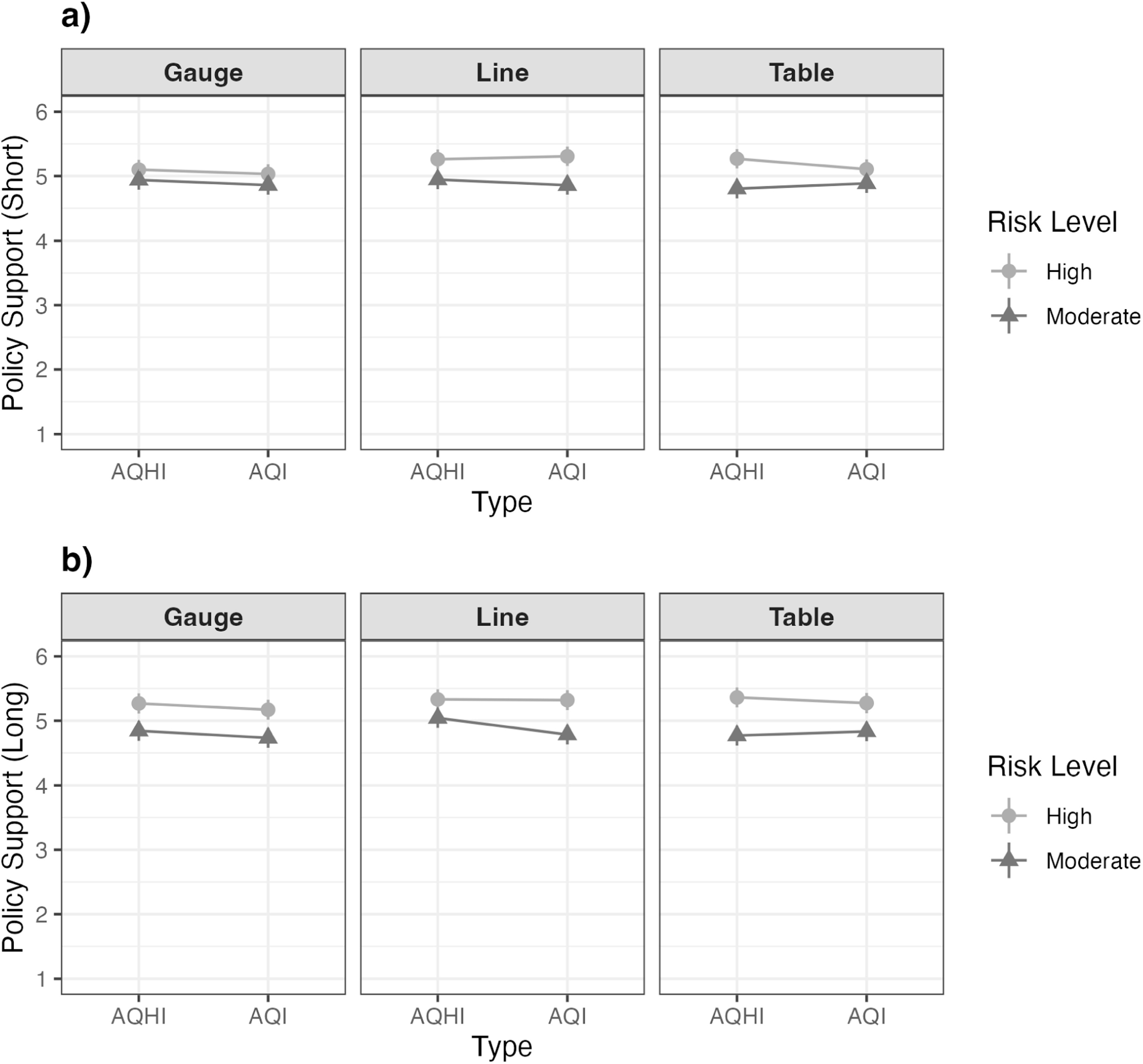
Post hoc comparison of policy support for various wildfire smoke exposure mitigation measures across the visual format, index type and risk level. (a) Short-term exposure scenario. (b) Long-term exposure scenario.

Results of the ANOVA (See table A4) showed only main effects of risk level in both the short- and long-exposure scenarios for our policy support measure, which included support for multiple exposure mitigation policies like moving outdoor events indoors and was assessed using 6-point scales (1 = Extremely opposed to 6 = Extremely supportive). In the short-exposure scenario, those who had viewed infographics displaying a high-risk level reported higher support for these exposure mitigation policies (*M* = 5.18, *SE =* 0.03) than those who had viewed infographics displaying moderate-risk (*M* = 4.88, *SE* = 0.03), *t* (*df* = 2088) = 6.71, *p* < 0.001. In the long-exposure scenario, the difference between high-risk (*M* = 5.29, *SE* = 0.03) and moderate-risk (*M* = 4.83, *SE* = 0.03) groups also significantly differed *t* (*df* = 2088) = 9.95, *p* < 0.001. There were no other main effects or interaction effects for the policy support measure (See figure A3 in the Appendix).

### School closure support: Short-term and long-term exposure scenarios

#### 3.5. Short-exposure

Additionally, we measured the effects of the infographics on support for school or childcare closures during unhealthy air quality days (measured using a 6-point scale, 1 = Extremely opposed to 6 = Extremely supportive). Closures are adopted by some school districts during especially hazardous wildfire smoke events since classrooms sometimes lack adequate ventilation (Sugerman *et al* 2012, Holm *et al* 2021). In the short-exposure scenario, results of the ANOVA showed that there was a main effect of risk level for the school closure support measure (See table A5). As with the policy support measure, those in the high-risk group (*M* = 4.55, *SE* = 0.05) indicated greater support for school or childcare closures than those in the moderate-risk group (*M* = 4.18, *SE* = 0.05), *t* (*df* = 2087) = 5.74, *p* < 0.001.

**Figure A4.**
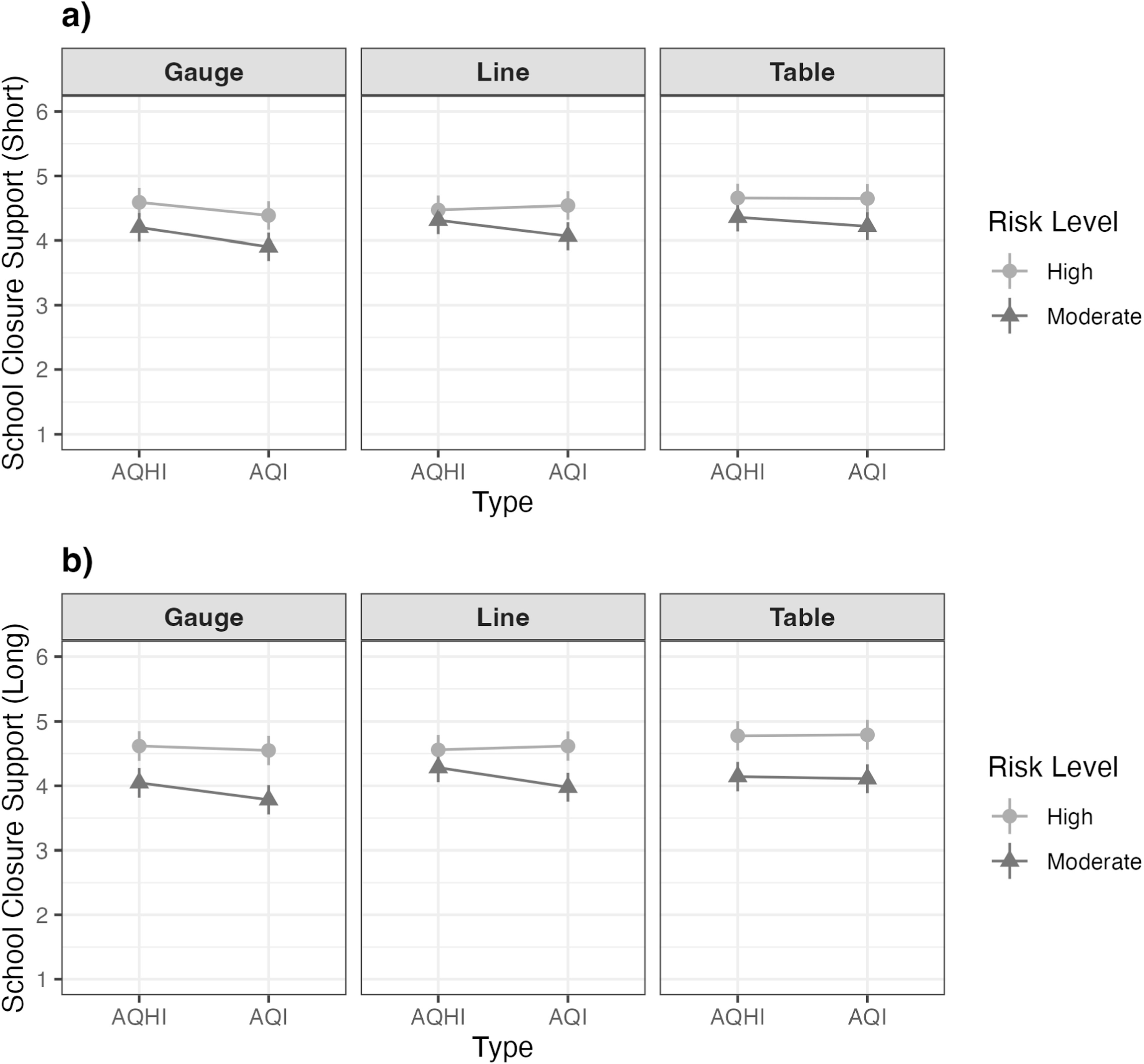
Post hoc comparison of support for school or childcare closures during unhealthy air quality days across the visual format, index type and risk level. (a) Short-term exposure scenario. (b) Long-term exposure scenario.

Main effects of index type and visual format were also observed in the short-exposure scenario (See figure A4 in the Appendix). Results by index type showed that those who had viewed the AQHI [1-11+] (*M* = 4.43, *SE* = 0.05) reported greater support for school or childcare closures than those who had viewed the AQI [0-500] (*M* = 4.30, *SE* = 0.05), *t* (*df* = 2087) = 2.13, *p* = 0.033. Across the three visual formats, participants who had viewed the table (*M* = 4.47, *SE* = 0.06) and line (*M* = 4.35, *SE* = 0.06) visuals reported greater support for school or childcare closure; support for closures did not differ significantly between these two visual formats, *t* (*df* = 2087) = 1.55, *p* = 0.121. Support for school or childcare closures among those viewing the gauge visual was lower (*M* = 4.27 *SE* = 0.06), and significantly differed from those viewing the table, *t* (*df* = 2087) = 2.52, *p* = 0.012; however, support did not differ between the gauge and line visual format groups, *t* (*df* = 2087) = 0.97, *p* = 0.331.

#### 3.6. Long-exposure

In the long-exposure scenario, results of the ANOVA (See table A5) showed that support for school or childcare closures (measured using the same 6-point scale) had a main effect of risk level such that the high-risk groups (*M* = 4.65, *SE* = 0.05) reported greater support than the moderate-risk groups (*M* = 4.06, *SE* = 0.05), *t* (*df* = 2083) = 8.85, *p* < 0.001. A main effect of visual format was also observed in the long-exposure scenario (See figure A4 in the Appendix). Across the three visual formats, participants who had viewed the table (*M* = 4.46, *SE* = 0.06) and line (*M* = 4.36, *SE* = 0.06) visuals reported greater support for school or childcare closures; support for closures did not differ significantly between these two visual formats, *t* (*df* = 2083) = 1.18, *p* = 0.240. Support for school or childcare closures among those viewing the gauge visual was lower (*M* = 4.25 *SE* = 0.06), and significantly differed from those viewing the table, *t* (*df* = 2083) = 2.50, *p* = 0.012; however, support did not differ between the gauge and line visual format groups, *t* (*df* = 2083) = 1.33, *p* = 0.183.

## 4. Discussion

### Discussion of key study findings

Children represent a sensitive group when it comes to wildfire smoke and exposures should likely be minimized and mitigated even when smoke levels are considered moderately risky (Hutchinson *et al* 2018, Holm *et al* 2021). Parents can play a large role in protecting their children from wildfire smoke, for example, by checking the local AQI, assessing the level of threat based on the AQI, and adopting risk-mitigating actions like reducing outdoor play time or using an indoor air purifier (McBride 2021, Slavik *et al* 2023). Since engagement with air quality information and concern about wildfire smoke influence the adoption of these actions (Hano *et al* 2020), disseminating effective smoke-risk communications is a key goal of governmental agencies aiming to promote smoke-safe behavior change (AirNow 2022). Yet, evaluations of current AQI communications and strategies for information dissemination have been limited.

In this experimental study with parents, we aimed to test which visuals and indices—adapted from common air quality communications in North America—work the best when wildfire smoke reaches moderate versus high levels, and during short-term versus long-term smoke events. We found that when infographics displayed high levels of wildfire smoke, parents seemed to recognize the risk whether exposures were framed as short-term or long-term, using any of the visuals and indices to communicate risk; all of them elicited similar levels of worry about smoke and action intentions. However, when moderate levels of wildfire smoke were displayed, using the AQHI [1-11+] in combination with line or gauge visuals led parents to report levels of worry and action intentions that more closely resembled parents’ responses at high levels of smoke. This finding may indicate that certain infographics could be used to motivate parents to protect their children from wildfire smoke exposure by helping them become more attuned to even moderate levels of smoke being hazardous to children’s health. Thus, this research may aid immediate practical applications. We hope that it also encourages further exploration of the underlying cognitive processes at work when people process AQI information, especially concerning the risks posed to both sensitive populations and the general public. Importantly, our work suggests that some agencies, such as in the US, may not be leveraging optimal methods of communicating about wildfire smoke risks.

Given children’s sensitivity to wildfire smoke, getting parents to consider ‘Moderate’ air quality days as potentially risky and motivating them to take actions against moderate *and* high levels of smoke may be desirable for public health. Our experimental findings indicate that using an index in the numeric style of the AQHI [1-11+], compared to the AQI [0-500], may increase parents’ perceptions of risk and promote smoke-safe behaviors during both short-term and longer-term wildfire smoke events. As the US-based AQI uses larger numbers, this finding may be explained by difficulties people have in differentiating large numbers and interpreting them appropriately (Parkman 1971, Boyce-Jacino *et al* 2022). Moreover, smaller numbers are more discriminable leading to quicker retrieval of their numerical values (Cohen Kadosh *et al* 2008, Peters *et al* 2008) and, thus, may be easier to apply towards individual decision-making.

The effectiveness of the 1-11+ index at supporting parents’ risk judgments of wildfire smoke, compared to the 0-500 index, is also supported by research on risk communication, which found that graphics with smaller denominators (e.g., AQHI of X out of 10) tend to be more easily and correctly interpreted by users compared to graphics displaying larger denominators (e.g., AQI of Y out of 500) (Ancker *et al* 2006, Fagerlin *et al* 2007). Furthermore, in prior studies of user preferences for air quality information, citizens have expressed a desire for simpler scales that are easier to understand and recall due to difficulties interpreting large numbers (Fish *et al* 2017, Ramírez *et al* 2019, Marfori *et al* 2020). Taken together, these prior works and our experimental results provide a compelling rationale for government agencies and communicators to consider adopting indices that use smaller numbers to convey air quality values like the AQHI.

Our results evaluating different visual formats of air quality information were a little more nuanced, presenting opportunities for further exploration. Given that prior reviews have pointed to visual aids as being potentially more effective for risk communication than tables (Ancker *et al* 2006, Fagerlin *et al* 2007, Garcia-Retamero and Cokely 2017), we expected the line and gauge visuals to significantly outperform the table with respect to parents’ worry, action intentions, and policy support. Yet, the effect of visual format was only significant sometimes and tables were not always suboptimal as reviewed below.

### Implications for environmental health policies and practice

Our findings provide some insights around choosing certain visual formats over others to advance specific communication goals. For example, if the goal is to influence risk perceptions and promote smoke-safe behavior change at ‘Moderate’ smoke levels, the line or gauge visuals appear to be more effective than a table. In our experiment, both the line and gauge visuals, when combined with the AQHI [1-11+], led to higher action intentions that more closely resembled those reported by parents during ‘High’ smoke levels. The line visual in combination with the AQHI [1-11+] elicited the most worry and the highest action intentions. An improved understanding of wildfire smoke risks and increased parental intention to take action, when exposed to the line visual and AQHI [1-11+], may be attributable to the ease with which individuals could use their mental number line (Izard and Dehaene 2008, Peters *et al* 2008) to interpret the displayed air quality value especially when presented with a visually consistent line representation. Although our study did not aim to investigate mechanisms of information processing or numeric cognition, the results suggest interesting follow-up studies to further elucidate potential effects of line-based visual communications on risk literacy.

On the other hand, if the goal is to educate parents about the need for school/childcare closures and gain support for this measure, a table appeared most effective based on our findings. School/childcare closures are typically a last-resort policy measure adopted only during prolonged and/or hazardous wildfire smoke events. This is because of disruptions to parents’ work schedules, possible impacts on loss of income, as well as concerns about some families experiencing similar levels of inadequate ventilation in their homes as in their local schools (Sugerman *et al* 2012, Holm *et al* 2021). Consequently, it was unsurprising to find parents’ support for closures, on average, to be lower than the other policy items. Still, closures are sometimes deemed necessary in some school districts (Washington State Departments of Health and Ecology 2024). As tables are generally thought to improve users’ memory of precise risk information relative to visuals (Hawley *et al* 2008, Tait *et al* 2010), it is possible that parents who had viewed the table in the experiment were able to recall more detailed information from the infographic text and effectively apply it towards this policy decision. This explanation is also consistent with our understanding of how visuals are often processed quickly using simple visual cues, and thus, may not always facilitate contemplative or effortful decision-making (Garcia-Retamero and Cokely 2017, Padilla *et al* 2018). Nonetheless, these findings should be interpreted cautiously as our experimental infographics only explained 2%-5% of the variance for the policy support and support for closures measures. Thus, more research is needed to uncover key predictors of parents’ support for various wildfire smoke risk-mitigation measures and policies.

### Study strengths and limitations

Key methodological strengths of this research include using experimental methods to directly compare different infographics adapted from real-life air quality communications, employing psychometrically reliable indices as dependent measures, and collecting data from a large, diverse sample of parents as study participants.

One notable limitation of this study is that observed effects may not have been solely attributable to the experimental conditions we tested (i.e., visual formats, index types, and risk levels). We performed a series of factorial analysis of covariance models to explore whether demographic variables predicted the outcome variables alongside the experimental conditions to which participants were assigned (See tables A7-A28). Indeed, prior research suggests that former experiences with wildfire and wildfire smoke, as well as regular engagement with air quality information, impact perceptions of risk and intentions to take risk-mitigating actions (D’Antoni 2019, Santana *et al* 2021, Berlin Rubin and Wong-Parodi 2022). Our study sample drew from a population located in jurisdictions where the occurrence of wildfires is becoming increasingly common and most of the parents are likely to have had some prior experience with wildfire smoke. In fact, our study’s recruitment period happened to coincide with a significant wildfire smoke season across many jurisdictions in North America. Future research could build on this work by comparing responses to air quality communications between populations with and without significant or repeated wildfire smoke exposures.

Relatedly, since our study sample focused exclusively on parents living in four jurisdictions in North America, our findings may lack global generalizability. Nonetheless, awareness of wildfire smoke health risks is not widespread even in regions where wildfires occur regularly and existing air quality communications frequently fail to reach vulnerable populations (Fish *et al* 2017, Ramírez *et al* 2019). Furthermore, children residing in wildfire-prone regions are at-risk of recurrent exposures to smoke throughout their growth and development, the health impacts of which are still poorly understood (Garcia *et al* 2021). As a result, we believe this sub-population—and their parents and caretakers—merit special consideration as a study population and as information consumers.

## 5. Conclusions

This research addresses a key knowledge gap related to effectively communicating about wildfire smoke risks to children’s health, especially in situations where air quality may not be perceived as hazardous by parents. Our study suggested that parents may have differentiated too much between moderate- and high-risk levels during a hypothetical wildfire smoke event given children’s sensitivity to wildfire smoke. Their worry and action intentions were meaningfully and significantly higher in the high-risk group than the moderate-risk group in both short-term and long-term exposure scenarios. However, in the short-exposure scenario, when shown the AQHI [1-11+] with either the line or gauge visuals, parents’ action intentions were more similar between moderate- and high-risk level groups. These results suggest that an AQHI-type of index may help parents be more attuned to even moderate levels of smoke-related air pollution being dangerous to children in short-exposure situations. Our findings contrast with common air quality communications in the US that use the AQI—rather than the AQHI—to inform people about wildfire-smoke risks. We anticipate that our research findings may be used by governments and officials to enhance existing air quality communication. Moreover, we hope that researchers will leverage this study to delve deeper into the use of visual communications to mitigate exposure to wildfire smoke and improve population health outcomes.

## Data Availability

The data that support the findings of this study will be openly available following an embargo at the following URL/DOI: https://doi.org/10.17605/OSF.IO/4J2S8. Data will be available from 1 July 2024.

## Appendices

**Table A1.** Results of ANOVA for the main effects and interactive effects of treatments on worry in the short and long-term exposure scenarios.

Model	Term	F	df	p	Partial eta^2^
Worry: Short-term (R^2^ = 0.28)	Intercept	24895.02	1, 2088	< 0.001	NA
	Index Type	19.13	1, 2088	< 0.001	0.009
	Risk Level	748.68	1, 2088	< 0.001	0.260
	Visual	3.64	2, 2088	0.026	0.003
	Index Type:Risk Level	9.65	1, 2088	0.002	0.005
	Index Type:Visual	1.21	2, 2088	0.297	0.001
	Risk Level:Visual	0.50	2, 2088	0.608	< 0.001
	3-way interaction	1.42	2, 2088	0.241	0.001
Worry: Long-term (R^2^ = 0.20)	Intercept	30310.67	1, 2088	< 0.001	NA
	Index Type	15.26	1, 2088	< 0.001	0.007
	Risk Level	470.51	1, 2088	< 0.001	0.18
	Visual	0.21	2, 2088	0.811	< 0.001
	Index Type:Risk Level	5.18	1, 2088	0.023	0.002
	Index Type:Visual	1.61	2, 2088	0.200	0.002
	Risk Level:Visual	2.87	2, 2088	0.057	0.003
	3-way interaction	2.55	2, 2088	0.078	0.002

**Table A2.** Results of ANOVA for the main effects and interactive effects of treatments on action intentions in the short and long-term exposure scenarios.

Model	Term	F	df	p	Partial eta^2^
Action Intentions: Short-term (R^2^ = 0.18)	Intercept	35608.40	1, 2088	< 0.001	NA
	Index Type	16.20	1, 2088	< 0.001	0.008
	Risk Level	411.59	1, 2088	< 0.001	0.160
	Visual	4.23	2, 2088	0.015	0.004
	Index Type:Risk Level	10.62	1, 2088	0.001	0.005
	Index Type:Visual	1.84	2, 2088	0.159	0.002
	Risk Level:Visual	0.43	2, 2088	0.653	< 0.001
	3-way interaction	6.03	2, 2088	0.002	0.006
Action Intentions: Long-term (R^2^ = 0.10)	Intercept	50884.15	1, 2088	< 0.001	NA
	Index Type	5.76	1, 2088	0.016	0.003
	Risk Level	212.01	1, 2088	< 0.001	0.09
	Visual	0.88	2, 2088	0.414	0.001
	Index Type:Risk Level	4.27	1, 2088	0.039	0.002
	Index Type:Visual	0.38	2, 2088	0.681	< 0.001
	Risk Level:Visual	0.10	2, 2088	0.906	< 0.001
	3-way interaction	2.18	2, 2088	0.113	0.002

**Table A3.** Means for each dependent variable by treatment group.

Condition	Index type	Risk level	Visual format	Worry (short)	Worry (long)	Action intention (short)	Action intention (long)	Policy support (short)	Policy support (long)	School closure (short)	School closure (long)
				Mean (standard error)							
1	AQI	Moderate	Table	3.94 (0.11)	4.56 (0.12)	3.50 (0.08)	3.92 (0.07)	4.89 (0.09)	4.83 (0.09)	4.22 (0.11)	4.11 (0.11)
2	AQI	Moderate	Line	3.70 (0.12)	4.42 (0.13)	3.24 (0.09)	3.81 (0.08)	4.86 (0.08)	4.78 (0.09)	4.07 (0.12)	3.98 (0.12)
3	AQI	Moderate	Gauge	3.66 (0.12)	4.59 (0.13)	3.12 (0.08)	3.75 (0.08)	4.86 (0.08)	4.73 (0.09)	3.90 (0.12)	3.78 (0.13)
4	AQI	High	Table	5.75 (0.09)	6.09 (0.08)	4.23 (0.06)	4.45 (0.05)	5.11 (0.08)	5.27 (0.08)	4.65 (0.10)	4.79 (0.11)
5	AQI	High	Line	5.61 (0.09)	5.97 (0.09)	4.27 (0.06)	4.45 (0.06)	5.31 (0.06)	5.32 (0.07)	4.54 (0.11)	4.62 (0.11)
6	AQI	High	Gauge	5.55 (0.09)	5.94 (0.09)	4.24 (0.06)	4.43 (0.05)	5.03 (0.08)	5.17 (0.08)	4.39 (0.11)	4.55 (0.11)
7	AQHI	Moderate	Table	4.15 (0.12)	4.69 (0.13)	3.50 (0.08)	3.93 (0.08)	4.80 (0.08)	4.77 (0.09)	4.36 (0.11)	4.14 (0.13)
8	AQHI	Moderate	Line	4.39 (0.11)	5.14 (0.12)	3.72 (0.07)	4.04 (0.07)	4.95 (0.08)	5.04 (0.08)	4.31 (0.12)	4.28 (0.12)
9	AQHI	Moderate	Gauge	4.14 (0.12)	4.88 (0.12)	3.53 (0.08)	4.00 (0.07)	4.94 (0.08)	4.84 (0.08)	4.20 (0.12)	4.05 (0.12)
10	AQHI	High	Table	5.87 (0.09)	6.25 (0.07)	4.36 (0.05)	4.52 (0.04)	5.27 (0.07)	5.36 (0.06)	4.66 (0.10)	4.78 (0.10)
11	AQHI	High	Line	5.71 (0.09)	6.05 (0.08)	4.30 (0.06)	4.45 (0.05)	5.26 (0.07)	5.33 (0.06)	4.47 (0.12)	4.56 (0.12)
12	AQHI	High	Gauge	5.56 (0.11)	6.01 (0.08)	4.17 (0.06)	4.39 (0.05)	5.10 (0.07)	5.27 (0.07)	4.59 (0.11)	4.62 (0.11)

**Table A4.** Results of ANOVA for the main effects and interactive effects of treatments on policy support in the short and long-term exposure scenarios.

Model	Term	F	df	p	Partial eta^2^
Policy Support: Short-term (R^2^ = 0.03)	Intercept	51694.44	1, 2088	< 0.001	NA
	Index Type	1.03	1, 2088	0.310	< 0.001
	Risk Level	45.03	1, 2088	< 0.001	0.02
	Visual	2.16	2, 2088	0.115	0.002
	Index Type:Risk Level	0.14	1, 2088	0.708	< 0.001
	Index Type:Visual	0.12	2, 2088	0.886	< 0.001
	Risk Level:Visual	2.24	2, 2088	0.107	0.002
	3-way interaction	1.59	2, 2088	0.204	0.002
Policy Support: Long-term (R^2^ = 0.05)	Intercept	49314.82	1, 2088	< 0.001	NA
	Type	3.36	1, 2088	0.067	0.002
	Level	98.98	1, 2088	< 0.001	0.05
	Graphic	2.15	2, 2088	0.117	0.002
	Type:Level	0.15	1, 2088	0.696	< 0.001
	Type:Graphic	0.66	2, 2088	0.519	< 0.001
	Level:Graphic	0.49	2, 2088	0.610	< 0.001
	3-way interaction	1.61	2, 2088	0.200	0.002

**Table A5.** Results of ANOVA for the main effects and interactive effects of treatments on support for school closures in the short and long-term exposure scenarios.

Model	Term	F	df	p	Partial eta^2^
School Closure Support: Short-term (R^2^ = 0.02)	Intercept	18026.25	1, 2087	< 0.001	NA
	Index Type	4.54	1, 2087	0.033	0.002
	Risk Level	32.95	1, 2087	< 0.001	0.02
	Visual	3.22	2, 2087	0.040	0.003
	Index Type:Risk Level	1.96	1, 2087	0.161	0.001
	Index Type:Visual	0.76	2, 2087	0.469	0.001
	Risk Level:Visual	0.28	2, 2087	0.755	< 0.001
	3-way interaction	0.28	2, 2087	0.758	< 0.001
School Closure Support: Long-term (R^2^ = 0.04)	Intercept	16781.69	1, 2083	< 0.001	NA
	Index Type	2.17	1, 2083	0.141	0.001
	Risk Level	78.28	1, 2083	< 0.001	0.04
	Visual	3.13	2, 2083	0.044	0.003
	Index Type:Risk Level	2.25	1, 2083	0.133	0.001
	Index Type:Visual	0.49	2, 2083	0.616	< 0.001
	Risk Level:Visual	1.04	2, 2083	0.352	0.001
	3-way interaction	0.46	2, 2083	0.632	< 0.001

### Checking for failures of random assignment

We performed a series of one-way analysis of variance (for metric variables) and analysis of deviance (for binary variables) to check for failures of random assignment on our demographic measures. We examined the overall significance of the F tests (for the analysis of variance models) and likelihood ratio tests (for the analysis of deviance models) to determine whether there was a significant failure of random assignment across study conditions. For location, which is a four-level categorical variable, we broke the variable down into a series of binary comparisons for modeling (e.g., British Columbia versus Oregon, Oregon versus California, etc). The table below shows the results of these analyses. There were no significant effects of the conditions on demographics in these models, suggesting no failures of random assignment.

**Table A6.** Analysis of variance/deviance results checking for failures of random assignment.

Demographic	F / Deviance statistic	df	p-value
Age (metric)	0.79	11, 2088	0.650
Income (metric)	1.27	11, 2021	0.238
Gender (binary)	14.63	2059	0.200
Bachelors versus Not (Binary)	12.06	2088	0.359
Parent or Child with Health Condition versus Not (Binary)	12.84	2088	0.304
Washington versus Oregon (Binary)	10.23	1049	0.510
Washington versus California (Binary)	7.44	1064	0.762
Washington versus British Columbia (Binary)	2.93	1025	0.992
Oregon versus California (Binary)	4.43	1051	0.956
Oregon versus British Columbia (Binary)	3.29	1012	0.986
California versus British Columbia (Binary)	3.42	1027	0.984

### Including covariates in models of each outcome variable

We performed a series of factorial analysis of covariance models to examine whether demographic variables predicted the outcome variables alongside the experimental conditions to which participants were assigned. In this section, we focus just on highlighting any significant effects of demographic characteristics on the outcome measures. For binary variables with significant effects (e.g., whether a participant has a Bachelor’s degree or not), we report the marginal means to compare the groups. For the location variable, when there are significant effects, we report the marginal means as well as a series of post-hoc comparisons between each location. For metric variables (e.g., income) we report a regression slope as an unstandardized coefficient *b*, along with the standard error of the slope and the p-value to demonstrate the direction of the effect highlighted in the analysis of covariance tables.

#### Worry: short-term

**Table A7.** Analysis of covariance results for worry in the short-term context (adjusted *R* ^2^ = 0.30).

Model Term	F	df	p-value	Partial eta^2^
Intercept	1000.30	1, 1989	< 0.001	NA
Index Type	24.52	1, 1989	< 0.001	0.01
Risk Level	721.48	1, 1989	< 0.001	0.27
Visual	3.93	2, 1989	0.020	0.004
Health Condition	62.76	1, 1989	< 0.001	0.03
Gender (binary)	8.33	1, 1989	0.004	0.004
Bachelor’s Degree	3.54	1, 1989	0.060	0.002
Family Income	0.01	1, 1989	0.932	< 0.001
Age	0.53	1, 1989	0.465	< 0.001
Location	7.41	3, 1989	< 0.001	0.01
Index Type:Risk Level	8.57	1, 1989	0.003	0.004
Index Type:Visual	0.83	2, 1989	0.434	0.001
Risk Level:Visual	0.13	2, 1989	0.877	< 0.001
3-way interaction	1.41	2, 1989	0.246	0.001

Women (M = 4.87, SE = 0.04) had greater worry in the short-term context than men (M = 4.68, SE = 0.05). Those having a health condition or a child with a condition (M = 5.03, SE = 0.04) had greater worry in the short-term context than those without a health condition or a child with a condition (M = 4.52, SE = 0.05). The location effects are detailed in the tables below.

**Table A8.** Marginal means for location in the short-term worry model.

	Marginal mean	SE
British Columbia	4.62	0.07
California	5.02	0.06
Oregon	4.73	0.06
Washington	4.73	0.06

**Table A9.** Location post-hoc pairwise comparisons in the short-term worry model.

Contrast	Estimate	SE	df	t-ratio	p-value
BC—CA	−0.40	0.09	1989	−4.82	< 0.001
BC—OR	−0.11	0.09	1989	−1.21	0.624
BC—WA	−0.12	0.09	1989	−1.30	0.561
CA—OR	0.29	0.09	1989	3.23	0.007
CA—WA	0.29	0.09	1989	3.27	0.006
OR—WA	−0.01	0.09	1989	−0.07	0.999

Tukey’s HSD p-value adjustment applied to adjust for multiple comparisons.

#### Worry: long-term

**Table A10.** Analysis of covariance results for worry in the long-term context (adjusted *R* ^2^ = 0.22).

Model term	F	df	p-value	Partial eta^2^
Intercept	1261.38	1, 1989	< 0.001	NA
Index Type	21.38	1, 1989	< 0.001	0.01
Risk Level	445.23	1, 1989	< 0.001	0.18
Visual	0.22	2, 1989	0.802	< 0.001
Health Condition	52.41	1, 1989	< 0.001	0.03
Gender (binary)	8.59	1, 1989	0.003	< 0.001
Bachelor’s Degree	4.30	1, 1989	0.038	0.002
Family Income	5.64	1, 1989	0.018	0.003
Age	0.02	1, 1989	0.895	< 0.001
Location	5.81	3, 1989	0.001	0.009
Index Type:Risk Level	5.16	1, 1989	0.023	0.003
Index Type:Visual	1.48	2, 1989	0.227	0.001
Risk Level:Visual	1.76	2, 1989	0.172	0.002
3-way interaction	2.84	2, 1989	0.059	0.003

Women (M = 5.43, SE = 0.04) had greater worry in the long-term context than men (M = 5.23, SE = 0.05). Those having a health condition or a child with a condition (M = 5.56, SE = 0.04) had greater worry in the long-term context than those without a health condition or a child with a condition (M = 5.10, SE = 0.05). Those with a Bachelor’s degree or greater had higher worry (M = 5.40, SE = 0.05) than those without a Bachelor’s degree (M = 5.26, SE = 0.05). Income had a negative slope such that those with higher income had slightly lower worry than those with lower income, b = −0.02, SE = 0.01, p = 0.018. The location effects are detailed in the tables below.

**Table A11.** Marginal means for location in the long-term worry model.

	Marginal mean	SE
British Columbia	5.17	0.07
California	5.54	0.06
Oregon	5.29	0.07
Washington	5.31	0.06

**Table A12.** Location post-hoc pairwise comparisons in the long-term worry model.

Contrast	Estimate	SE	df	t-ratio	p-value
BC - CA	−0.37	0.09	1989	−4.05	< 0.001
BC—OR	−0.11	0.09	1989	−1.21	0.619
BC—WA	−0.14	0.09	1989	−1.56	0.405
CA—OR	0.26	0.09	1989	2.80	0.027
CA—WA	0.23	0.09	1989	2.57	0.050
OR - WA	−0.03	0.09	1989	−0.32	0.989

Tukey’s HSD p-value adjustment applied to adjust for multiple comparisons.

#### Action intentions: short-term

**Table A13.** Analysis of covariance results for action intentions in the short-term context (adjusted *R* ^2^ = 0.22).

Model term	F	df	p-value	Partial eta^2^
Intercept	1434.80	1, 1989	< 0.001	NA
Index Type	19.59	1, 1989	< 0.001	0.01
Risk Level	399.94	1, 1989	< 0.001	0.17
Visual	3.85	2, 1989	0.021	0.004
Health Condition	67.29	1, 1989	< 0.001	0.03
Gender (binary)	24.87	1, 1989	< 0.001	0.01
Bachelor’s Degree	5.16	1, 1989	0.023	0.003
Family Income	1.91	1, 1989	0.167	0.001
Age	2.04	1, 1989	0.154	0.001
Location	5.97	3, 1989	< 0.001	0.009
Index Type:Risk Level	11.37	1, 1989	0.001	0.006
Index Type:Visual	1.00	2, 1989	0.367	0.001
Risk Level:Visual	1.06	2, 1989	0.348	0.001
3-way interaction	6.39	2, 1989	0.002	0.006

Women (M = 3.91, SE = 0.03) had greater short-term action intentions than men (M = 3.70, SE = 0.03). Those having a health condition or a child with a condition (M = 3.98, SE = 0.03) had greater short-term action intentions than those without a health condition or a child with a condition (M = 3.63, SE = 0.03). Education had an effect such that those with a Bachelor’s degree or greater had higher short-term action intentions (M = 3.86, SE = 0.03) than those without a Bachelor’s degree (M = 3.75, SE = 0.03). The location effects are detailed in the tables below.

**Table A14.** Marginal means for location in the short-term action intentions model.

	Marginal mean	SE
British Columbia	3.65	0.04
California	3.87	0.04
Oregon	3.84	0.04
Washington	3.86	0.04

**Table A15.** Location post-hoc pairwise comparisons in the short-term action intentions model.

Contrast	Estimate	SE	df	t-ratio	p-value
BC—CA	−0.22	0.06	1989	−3.72	0.001
BC—OR	−0.19	0.06	1989	−3.09	0.011
BC—WA	−0.21	0.06	1989	−3.53	0.002
CA—OR	0.03	0.06	1989	0.56	0.943
CA—WA	0.01	0.06	1989	0.23	0.996
OR—WA	−0.02	0.06	1989	−0.36	0.984

Tukey’s HSD p-value adjustment applied to adjust for multiple comparisons.

#### Action intentions: long-term

**Table A16.** Analysis of covariance results for action intentions in the long-term context (adjusted *R* ^2^ = 0.15).

Model term	F	df	p-value	Partial eta^2^
Intercept	2036.66	1, 1989	< 0.001	NA
Index Type	9.49	1, 1989	0.002	0.005
Risk Level	201.44	1, 1989	< 0.001	0.09
Visual	0.69	2, 1989	0.500	0.001
Health Condition	70.32	1, 1989	< 0.001	0.03
Gender (binary)	31.79	1, 1989	< 0.001	0.02
Bachelor’s Degree	1.71	1, 1989	0.191	0.001
Family Income	2.89	1, 1989	0.089	0.001
Age	1.55	1, 1989	0.213	0.001
Location	4.36	3, 1989	0.005	0.007
Index Type:Risk Level	4.98	1, 1989	0.026	0.003
Index Type:Visual	0.17	2, 1989	0.846	< 0.001
Risk Level:Visual	0.03	2, 1989	0.969	< 0.001
3-way interaction	2.48	2, 1989	0.084	0.002

Women (M = 4.24, SE = 0.03) had greater action intentions in the long-term context than men (M = 4.02, SE = 0.03). Those having a health condition or a child with a condition (M = 4.29, SE = 0.02) had greater action intentions in the long-term context than those without a health condition or a child with a condition (M = 3.97, SE = 0.03). The location effects are detailed in the tables below.

**Table A17.** Marginal means for location in the long-term action intentions model.

	Marginal mean	SE
British Columbia	4.01	0.04
California	4.17	0.04
Oregon	4.16	0.04
Washington	4.19	0.04

**Table A18.** Location post-hoc pairwise comparisons in the long-term action intentions model.

Contrast	Estimate	SE	df	t-ratio	p-value
BC - CA	−0.16	0.05	1989	−2.92	0.019
BC—OR	−0.14	0.05	1989	−2.64	0.042
BC—WA	−0.17	0.05	1989	−3.27	0.006
CA—OR	0.01	0.05	1989	0.22	0.996
CA—WA	−0.02	0.05	1989	−0.33	0.987
OR - WA	−0.03	0.05	1989	−0.57	0.941

Tukey’s HSD p-value adjustment applied to adjust for multiple comparisons.

#### Policy composite: short-term

**Table A19.** Analysis of covariance results for policy support in the short-term context (adjusted *R* ^2^ = 0.09).

Model term	F	df	p-value	Partial eta^2^
Intercept	1883.99	1, 1989	< 0.001	NA
Index Type	1.57	1, 1989	0.210	0.001
Risk Level	42.40	1, 1989	< 0.001	0.02
Visual	1.99	2, 1989	0.137	0.002
Health Condition	44.83	1, 1989	< 0.001	0.02
Gender (binary)	70.42	1, 1989	< 0.001	0.03
Bachelor’s Degree	0.03	1, 1989	0.864	< 0.001
Family Income	3.05	1, 1989	0.081	0.002
Age	0.65	1, 1989	0.419	< 0.001
Location	3.33	3, 1989	0.019	0.005
Index Type:Risk Level	0.14	1, 1989	0.704	< 0.001
Index Type:Visual	0.44	2, 1989	0.641	< 0.001
Risk Level:Visual	1.92	2, 1989	0.147	0.002
3-way interaction	2.11	2, 1989	0.122	0.002

Women (M = 5.17, SE = 0.03) had greater short-term policy support than men (M = 4.77, SE = 0.03). Those having a health condition or a child with a condition (M = 5.12, SE = 0.03) had greater short-term policy support than those without a health condition or a child with a condition (M = 4.82, SE = 0.03). The location effects are detailed in the tables below.

**Table A20.** Marginal means for location in the short-term policy support model.

	Marginal mean	SE
British Columbia	4.86	0.05
California	4.95	0.04
Oregon	5.04	0.05
Washington	5.03	0.04

**Table A21.** Location post-hoc pairwise comparisons in the short-term policy support model.

Contrast	Estimate	SE	df	t-ratio	p-value
BC—CA	−0.09	0.06	1989	−1.47	0.453
BC—OR	−0.18	0.07	1989	−2.77	0.029
BC—WA	−0.17	0.06	1989	−2.73	0.032
CA—OR	−0.09	0.06	1989	−1.35	0.533
CA—WA	−0.08	0.06	1989	−1.27	0.585
OR—WA	0.01	0.06	1989	0.13	0.999

Tukey’s HSD p-value adjustment applied to adjust for multiple comparisons.

#### Policy composite: long-term

**Table A22.** Analysis of covariance results for policy support in the long-term context (adjusted *R* ^2^ = 0.10).

Model Term	F	df	p-value	Partial eta^2^
Intercept	1756.72	1, 1989	< 0.001	NA
Index Type	5.06	1, 1989	0.025	0.003
Risk Level	98.82	1, 1989	< 0.001	0.05
Visual	1.72	2, 1989	0.179	0.002
Health Condition	41.69	1, 1989	< 0.001	0.02
Gender (binary)	47.03	1, 1989	< 0.001	0.02
Bachelor’s Degree	0.51	1, 1989	0.477	< 0.001
Family Income	5.07	1, 1989	0.025	0.003
Age	0.67	1, 1989	0.413	< 0.001
Location	2.25	3, 1989	0.080	0.003
Index Type:Risk Level	0.25	1, 1989	0.620	< 0.001
Index Type:Visual	0.23	2, 1989	0.798	< 0.001
Risk Level:Visual	0.05	2, 1989	0.955	< 0.001
3-way interaction	1.68	2, 1989	0.187	0.002

Women (M = 5.17, SE = 0.03) had greater policy support in the long-term context than men (M = 4.84, SE = 0.04). Those having a health condition or a child with a condition (M = 5.16, SE = 0.03) had greater policy support in the long-term context than those without a health condition or a child with a condition (M = 4.85, SE = 0.04). There was a positive slope for income such that as income increased policy support also increased, b = 0.02, SE = 0.01, p = 0.025.

#### School closure: short-term

**Table A23.** Analysis of covariance results for support for school closures in the short-term context (adjusted *R* ^2^ = 0.07).

Model term	F	df	p-value	Partial eta^2^
Intercept	839.91	1, 1988	< 0.001	NA
Index Type	5.60	1, 1988	0.018	0.003
Risk Level	30.48	1, 1988	< 0.001	0.02
Visual	3.09	2, 1988	0.046	0.003
Health Condition	17.87	1, 1988	< 0.001	0.009
Gender (binary)	2.63	1, 1988	0.105	0.001
Bachelor’s Degree	0.67	1, 1988	0.416	< 0.001
Family Income	22.30	1, 1988	< 0.001	0.01
Age	0.03	1, 1988	0.856	< 0.001
Location	16.43	3, 1988	< 0.001	0.02
Index Type:Risk Level	1.87	1, 1988	0.172	0.001
Index Type:Visual	0.43	2, 1988	0.650	< 0.001
Risk Level:Visual	0.41	2, 1988	0.663	< 0.001
3-way interaction	0.24	2, 1988	0.786	< 0.001

Those having a health condition or a child with a condition (M = 4.46, SE = 0.04) had greater support for school closures in the short-term context than those without a health condition or a child with a condition (M = 4.18, SE = 0.05). There was a negative slope for income such that those with higher income were less supportive of school closures in the short-term context, b = −0.05, SE = 0.01, p < 0.001. The location effects are detailed in the tables below.

**Table A24.** Marginal means for location in the school closure model in the short-term context.

	Marginal Mean	SE
British Columbia	3.95	0.07
California	4.59	0.07
Oregon	4.45	0.07
Washington	4.30	0.07

**Table A25.** Location post-hoc pairwise comparisons in the school closure model in the short-term context.

Contrast	Estimate	SE	df	t-ratio	p-value
BC—CA	−0.64	0.10	1988	−6.70	< 0.001
BC—OR	−0.50	0.10	1988	−5.12	< 0.001
BC—WA	−0.35	0.09	1988	−3.71	0.001
CA—OR	0.14	0.10	1988	1.47	0.458
CA—WA	0.29	0.09	1988	3.09	0.011
OR—WA	0.15	0.09	1988	1.60	0.378

Tukey’s HSD p-value adjustment applied to adjust for multiple comparisons.

#### School closure: long-term

**Table A26.** Analysis of covariance results for support for school closures in the long-term context (adjusted *R* ^2^ = 0.10).

Model Term	F	df	p-value	Partial eta^2^
Intercept	867.57	1, 1984	< 0.001	NA
Index Type	2.90	1, 1984	0.089	0.001
Risk Level	78.87	1, 1984	< 0.001	0.04
Visual	3.12	2, 1984	0.045	0.003
Health Condition	28.61	1, 1984	< 0.001	0.01
Gender (binary)	2.91	1, 1984	0.088	0.001
Bachelor’s Degree	1.24	1, 1984	0.266	0.001
Family Income	27.78	1, 1984	< 0.001	0.01
Age	1.69	1, 1984	0.194	0.001
Location	19.87	3, 1984	< 0.001	0.03
Index Type:Risk Level	1.95	1, 1984	0.162	0.001
Index Type:Visual	0.32	2, 1984	0.724	< 0.001
Risk Level:Visual	0.53	2, 1984	0.589	0.001
3-way interaction	0.21	2, 1984	0.808	< 0.001

Those having a health condition or a child with a condition (M = 4.48, SE = 0.04) had greater support for school closures in the long-term context than those without a health condition or a child with a condition (M = 4.11, SE = 0.05). There was a negative slope for income such that those with higher income were less supportive of school closures in the long-term context, b = −0.05, SE = 0.01, p < 0.001. The location effects are detailed in the tables below.

**Table A27.** Marginal means for location in the school closure model in the long-term context.

	Marginal mean	SE
British Columbia	3.90	0.07
California	4.65	0.07
Oregon	4.36	0.07
Washington	4.27	0.07

**Table A28.** Location post-hoc pairwise comparisons in the school closure model in the long-term context.

Contrast	Estimate	SE	df	t-ratio	p-value
BC—CA	−0.75	0.10	1984	−7.66	< 0.001
BC—OR	−0.46	0.10	1984	−4.57	< 0.001
BC—WA	−0.37	0.10	1984	−3.78	0.001
CA—OR	0.29	0.10	1984	2.98	0.016
CA—WA	0.38	0.10	1984	4.00	< 0.001
OR—WA	0.09	0.09	1984	0.94	0.781

Tukey’s HSD p-value adjustment applied to adjust for multiple comparisons.

### Correlations among dependent measures

The table below provides the Pearson *r* correlation coefficients between each of the dependent measures measured in the study.

**Table A29.** Pearson *r* correlations for each dependent measure used in the study.

	(1)	(2)	(3)	(4)	(5)	(6)	(7)	(8)
Worry Short (1)	1							
Worry Long (2)	0.77	1						
Actions Short (3)	0.71	0.68	1					
Actions Long (4)	0.58	0.72	0.77	1				
Policy Short (5)	0.37	0.42	0.52	0.59	1			
Policy Long (6)	0.45	0.55	0.59	0.73	0.71	1		
School Closure Short (7)	0.35	0.39	0.39	0.41	0.48	0.41	1	
School Closure Long (8)	0.41	0.51	0.47	0.52	0.38	0.53	0.77	1

All p-values < 0.001.

## Funding sources

Support for this research was provided by a grant from the National Institute of Environmental Health Sciences, National Institutes of Health (Grant Number: P2C ES033432). The work was also supported in part by the Banting Postdoctoral Fellowships program of Canada and the US National Science Foundation (# SES-2017651). The funders did not play a role in the study’s conceptualization, design, data collection, analysis, nor in the preparation of this manuscript.

## Ethics

The study received approval from the University of Oregon’s Institutional Review Board (ID#00000870).

## Data availability

Data and study materials are publicly available for download at https://doi.org/10.17605/OSF.IO/4J2S8.

## Conflict of interest

The authors declare no competing interests.
